# Syndromic Surveillance Models Using Web Data: The Case of Influenza in Greece and Italy Using Google Trends

**DOI:** 10.2196/publichealth.8015

**Published:** 2017-11-20

**Authors:** Loukas Samaras, Elena García-Barriocanal, Miguel-Angel Sicilia

**Affiliations:** ^1^ Computer Science Department University of Alcalá Alcalá de Henares (Madrid) Spain

**Keywords:** Google Trends, influenza, Web, syndromic surveillance, statistical correlation, forecast, ARIMA

## Abstract

**Background:**

An extended discussion and research has been performed in recent years using data collected through search queries submitted via the Internet. It has been shown that the overall activity on the Internet is related to the number of cases of an infectious disease outbreak.

**Objective:**

The aim of the study was to define a similar correlation between data from Google Trends and data collected by the official authorities of Greece and Europe by examining the development and the spread of seasonal influenza in Greece and Italy.

**Methods:**

We used multiple regressions of the terms submitted in the Google search engine related to influenza for the period from 2011 to 2012 in Greece and Italy (sample data for 104 weeks for each country). We then used the autoregressive integrated moving average statistical model to determine the correlation between the Google search data and the real influenza cases confirmed by the aforementioned authorities. Two methods were used: (1) a flu score was created for the case of Greece and (2) comparison of data from a neighboring country of Greece, which is Italy.

**Results:**

The results showed that there is a significant correlation that can help the prediction of the spread and the peak of the seasonal influenza using data from Google searches. The correlation for Greece for 2011 and 2012 was .909 and .831, respectively, and correlation for Italy for 2011 and 2012 was .979 and .933, respectively. The prediction of the peak was quite precise, providing a forecast before it arrives to population.

**Conclusions:**

We can create an Internet surveillance system based on Google searches to track influenza in Greece and Italy.

## Introduction

### Syndromic Surveillance on Influenza

Syndromic surveillance systems refer to monitoring of infectious diseases through data collection from various sources. This is accomplished by setting indicators and methods and publishing reports for early detection of an infectious disease. The desired result is to minimize the extensive spread in the population and take precautionary measures.

These systems operate both at national and international levels and provide useful data and guidelines to deal with various outbreaks of different pathogens and infections. Influenza is considered as an important example for syndromic surveillance and response.

At the international level, the World Health Organization (WHO) has launched the Global Influenza Program [1]. It provides support to member states for a more efficient coordination of national health systems and for the proper preparation against seasonal influenza outbreaks.

Special monitoring and laboratories projects are used, such as the Global Influenza Surveillance and Response System [2]. This surveillance system works under the Pandemic Influenza Preparedness Framework. It became effective after the May 24, 2011 adoption by the 64th World Health Assembly [3].

In Europe, the competent public health authorities use the European Influenza Surveillance Network (EISN) [4] that combines epidemiological and virological surveillance of influenza. The purpose of this network is to assist in public health decision making. It provides experts in European Union (EU) or European Economic Area member states with the information required for better assessment of influenza activity in Europe, resulting in the appropriate action. Finally, EISN’s goal is to contribute in reducing the costs associated with influenza in Europe. Epidemiological and virological influenza surveillance data are collected through the European Surveillance System [5].

As described in the Fact Sheet no. 211 (revised March 2003) of WHO [6], influenza is caused by a virus that mainly attacks specific parts of the human body, for example, nose, throat, bronchi, and rarely the lungs. The main symptoms are high fever, myalgia, headache and severe malaise, nonproductive cough, sore throat, and rhinitis. Severe health complications of influenza virus infection in susceptible individuals include pneumonia and eventually death. The influenza virus is easily transferred from person to person through air. It enters the body through the upper respiratory tract (nose or throat) when a person coughs or sneezes. A few days may pass before a patient’s symptoms are clearly recognized. The development of symptoms may not be clear on the first day; however, it can be recognized after 7 days. Upper respiratory tract infections affect 5% to 15% of the population. The estimated annual impact of severe illness cases on the population is 3 to 5 million; 250,000-500,000 of which die. The spread of the disease among the population is very quick, especially in crowded cases. The survival period of the virus outside the body can be further enhanced in cold and dry weather. Consequently, the seasonal epidemics in temperate areas occur in winter. Influenza mostly appears in the weakest portion of population; people older than 65 years or those with serious health problems such as lung diseases, diabetes, cancer, and kidney or heart problems.

The corresponding health costs are high. Only in the United States, large sums of money are directed to influenza treatment and hospitalization. The annual cost for the United States [7] is US $71 to 167 billion. In Russia [8], the government allocated 4 billion rubles (US $140 million) to buy the initial 43 million doses of vaccines to perform mass swine flu vaccinations in the year 2009. Russia planned to have 35.5 million doses before the end of this year.

### Related Research

Web data is now frequently being used in research conducted by scientists, and they show that the Internet may be an alternative source for collection of data that indicate the development of a syndromic disease using search engine queries. Eysenbach [9] and later also Ginsberg et al [10] examined the potential of Google search queries to *track influenza-like illness in a population*. The latter work uses data from the US Centers for Disease Control and Prevention (CDC) for influenza and linear regression methods to reveal statistical correlations and using the most significant keywords most searched into Web queries, constructs a kind of *flu score* and examines its statistical correlation to the data from the national disease center. The results of both studies showed that Web queries indicate the public interest and follow the actual spread of influenza in Canada or the United States, respectively.

Syndromic surveillance relies on the real-time use of information about the population to identify health issues of concern, and it is the current tool used by public health authorities to address them before they become epidemics. Consequently, a syndromic surveillance system implements a variety of outbreak detection algorithms, requiring a good understanding of the strengths and limitations of various detection techniques and their applicability. For example, Ping et al used data which were available via the Web and from physicians’ databases [11]. 

Our group has conducted similar research for another infectious disease (scarlet fever) in the United Kingdom. We used Web data [12] from Google Insights for Search [13], which is now merged with Google Trends [14]. We correlated data using linear regression techniques and exploiting the benefits and properties of the gamma distribution.

Hopkins University researchers in Baltimore, United States, find *Google Flu Trends* a powerful early warning system for emergency departments (EDs) [15]. This study [16] was a retrospective observational study of patients with symptoms of influenza. These patients presented to urban academic EDs in Baltimore, Maryland. The annual visits were 60,000 for adults and 24,000 for pediatric cases. The period of the visits was 21 months, from January 25, 2009 to October 3, 2010.

According to the CDC’s definition of fever and cough or sore throat, the researchers used the CDC’s traditional surveillance methods reporting system from January 25, 2009 to October 18, 2009 and an ED electronic reporting system (from October 18, 2009 to October 3, 2010).

Google Flu Trends weekly data were collected for Baltimore, Maryland. They also collected data from ED, CDC-reported standardized influenza-like illness (ILI) data, and influenza data confirmed by laboratories.

The data were analyzed separately for adult and pediatric cases and correlated to the Google data using cross-correlation functions. The conclusions of this study were that city-level Google Flu Trends shows strong correlation with influenza cases and EDs’ ILI visits, validating its use as an ED surveillance tool. Google Flu Trends correlated with several pediatric ED crowding measures and those for low-acuity adult patients.

Two other research studies were conducted using the autoregressive integrated moving average (ARIMA) model, by Dugas et al (*Influenza forecasting with Google Flu Trends*) [17] and by Preis and Moat (*Adaptive now casting of influenza outbreaks using Google searches*) [18]. This model combines autoregression and moving average model into one, as will be described further in the Methods section of this paper.

Recent research [19,20] has also shown the potential use of the Google Search engine to track influenza. The research on the use of Google reveals the strong statistical correlation between Google searches and influenza, even though there are variations over geographic location and time limits of this kind of estimation.

In 2017, several studies [21] investigated the use of the Internet to reveal the connections between the social activity on the Internet and the development of various diseases and mental disorders or problems such as norovirus epidemics, breast cancer, depression, cannabis dispensaries, Zika virus, Ebola virus, other drugs, Lyme borreliosis, whiplash syndrome, etc, using Google, Twitter, or other Internet sources.

In this study, we examine the development and the spread of seasonal influenza in Greece and Italy. Our goal is to define the correlation (and finally accomplish prediction patterns) between data from Google Trends and data collected by the Hellenic Center for Disease Control and Prevention (KEELPNO) [22] for Greece and data from the European Center for Disease and Control (ECDC) for Italy. The case as it pertains to Greece is quite different from those on previous studies. This is because, using the data from Google Trends for the term *influenza,* the correlation coefficient (Pearson *r*) is very low at .554 *.* This means that we cannot create estimation and prediction patterns using this keyword. For the Greek word of influenza (*Γρίπη* in Greek language), there are some data for years 2011 and 2012, but they are not very reliable. The following Methods and Results sections address the aforementioned problem.

## Methods

### Data Used

For the purposes of this study, we used datasets as follows:

- Weekly data for ILI from the sentinel system of KEELPNO for the years 2011 to 2012 (105 weeks), for which we could find data. In Greece, through the sentinel system, the influenza activity is monitored on a weekly basis. This system consists of three basic networks: (1) from selected health units of the largest social security organization (IKA) [23], which covers over 5,530,000 people and provides over 830,000 pensions; (2) from a network of selected private physicians; and (3) from a network of selected health centers and regional doctors’ offices. From these three networks, data are collected on a weekly basis regarding the number of patients’ visits for any cause, as well as the number of patients’ visits because of ILI based on the current EU case definition [24]. Data analysis includes weighting based on the resident population of classification of territorial units for statistics (NUTS I) geographical regions and rural and urban areas and produces the number of ILI cases per 1000 visits for every week of the year, for the total of the country. The collection and analysis of the data follow the ISO 8601 standard [25] (Greece ELOT EN 28601 standard [26]).
- We also used data from Italy for the same period and compared the results between the two countries. To get the ILI rates for Italy, we used the weekly reports from ECDC [27]. The ECDC calculates ILI rates per 100,000 people based on sentinel systems of the European member states.
- We used weekly data from the Google Trends for Greece and Italy, using C# programming code (see Multimedia Appendix 1).

Google Trends analyzes [28] a portion of Google Web searches to compute how many searches have been done for the terms entered in the search engine, relative to the total number of searches done on Google over time. This analysis indicates the likelihood of a random user to search for a search term from a certain location at a certain time. This system designates a certain threshold of traffic for search terms, so that these with low volume do not appear. Google Trends also eliminates repeated queries from a single user over a short period of time, so that the level of interest is not artificially impacted by these types of queries.

To calculate the popularity of a searched term among users in a certain geographical location (eg, country) and in a certain period, Trends examines a percentage of all searches for the specified term within the same time and location parameters. The results are then shown on a graph plotted on a scale from 0 to 100. The same information is also displayed graphically by the geographic heat map.

In our case, we must deal with the problem that for the term *influenza,* there is not appropriate search volume for Greece, as mentioned in the Introduction. For the Greek equivalent keyword (*Γρίπη*), a search volume exists but with low correlation or correlation below .90, as this is shown in Table 1.

The solution to this problem is to perform searches for separate keywords related to the term *influenza.* So, we downloaded data from Google Trends for the following keywords: *γριπη*, *πυρετος*, *βηχας*, *πονοκεφαλος*, *πονολαιμος*, *φαρυγγιτιδα,* and *αντιβιωση.* These keywords correspond to the following English terms: *influenza*, *fever*, cough, *headache, sore throat*, *pharyngitis,* and *antibiotics,* respectively. All these keywords refer to the symptoms and treatment of influenza.

Our next task was to determine whether we can use one of these or all together, creating a *flu score*. Using Statistical Package for the Social Sciences (SPSS) version 20 (IBM Corp), we wrote a command syntax code (see Multimedia Appendix 1) to perform multiple regressions by taking each Google dataset as the independent variable and data from the sentinel system of the KEELPNO as the dependent variable.

The results of the multiple regressions are shown in Table 1. In the table, x1, x2, x3, x4, x5, x6, and x7 represent the seven keywords or datasets of the independent variable (Google searches), whereas Y is the dependent variable (predicted variable, influenza ILI rates).

**Table 1:** Regressions of separate keywords (year 2011).

Keyword	English term	Variable	*r*	*R*^2^	Standard error	Constant	Coefficient
γριπη	Influenza	x1	.888765	.789903	0.00996	.008855	.539551
πυρετος	Fever	x2	.655427	.429584	0.01641	−.03361	2.747708
βηχας	Cough	x3	.658775	.433984	.01634	−.01984	2.03161
πονοκεφαλος	Headache	x4	.007578	.000057	0.02172	.019887	.034104
πονολαιμος	Sore throat	x5	.242802	.058953	0.02107	.01486	.22728
φαρυγγιτιδα	Pharyngitis	x6	.340787	.116136	0.02042	.005607	.708454
αντιβιωση	Antibiotics	x7	.327644	.107351	0.02052	−.01148	1.596785

As shown in Table 1, no regression yields a correlation factor (*r*) >.90.

In our case, we decided to combine all the keywords and create a flu score.

To obtain the data from Google Trends, we used Visual Studio 2012 Ultimate and Visual C# as the programming language.

### Methods Employed

Our goal was to construct prediction models based on the ARIMA model, previously used by other researchers, in specific data from Greece and Italy for influenza, describing two cases: using a flu score for Greece and without it for Italy.

**Figure 1:**
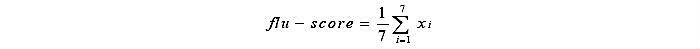
Flu-score equation.

We examined two different cases, both based on ARIMA models. The assumptions are based on that we can create a flu score from different keywords searched by people on the Internet. This score consists of the separate keywords, and it is the aggregation of them. In terms of statistics, this score is the average of all the values of each separate keyword, as shown in the following Figure 1 where x_i_=the values of each independent variable (x1, x2, x3, x4, x5, x6, and x7).

The first case assumes that this score can be created and used (the case of Greece), whereas the other assumes that there is enough and reliable data from the Internet that can be safely used. In that case, we used data from a neighboring country using the keyword *influenza* from Google Trends (the case of Italy). Finally, we compared the two cases, having as criteria the statistical correlation coefficient *r* and the results of the ARIMA models. We conducted experiments with the ARIMA model with small parameters (parameters from 0-3), as the data sample is relatively small, and the ARIMA (1, 0, 0) model was found the only one to be statistically significant at level of *P*<.05 (two-tailed).

### The Case of Greece Using a “Flu Score” and the ARIMA Model

#### Model Estimation for Year 2011

After creating the flu score, we used the model ARIMA (1, 0, 0) [29], a model also known as the Box-Jenkins model [30]. We used lags for the independent variable (Google data). This is a model that combines an autoregression and a moving average model.

Lags of the differenced series appearing in the forecasting equation are called autoregressive terms, lags of the forecast errors are called moving average terms, and a time series, which needs to be differenced to be made stationary, is said to be an integrated version of a stationary series. The ARIMA models are, in theory, the most general class of models for forecasting a time series, which can be stationarized by transformations such as differencing and logging.

A nonseasonal ARIMA model is classified as an ARIMA (p, d, q) model, where p is the number of autoregressive terms, d is the number of nonseasonal differences, and q is the number of lagged forecast errors in the prediction equation.

In more detail, the above parameters can be analyzed as follows:

p stands for the number of autoregressive orders in the model. Autoregressive orders specify which previous values from the series are used to predict current values. For example, an autoregressive order of 2 specifies that the value of the series two-time periods in the past be used to predict the current value.

d specifies the order of differencing applied to the series before estimating models. Differencing is necessary when trends are present (series with trends are typically nonstationary and ARIMA modeling assumes stationarity) and is used to remove their effect. The order of differencing corresponds to the degree of series trend—first-order differencing accounts for linear trends, second-order differencing accounts for quadratic trends, and so on.

Finally, q means the number of moving average orders in the model. Moving average orders specify how deviations from the series mean for previous values are used to predict current values. For example, moving average orders of 1 and 2 specify that deviations from the mean value of the series from each of the last two-time periods be considered when predicting current values of the series.

In our model, we do not use nonseasonal differences, as we examine a single period of a year, which means there is no seasonality inside the same year, and the peak occurs only once. This model is a special case of an ARIMA model (autoregressive moving average [ARMA] model).

**Figure 2:**
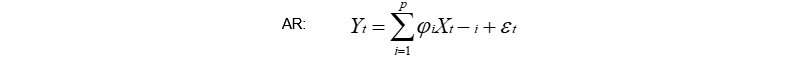
Autoregression equation.

**Figure 3:**
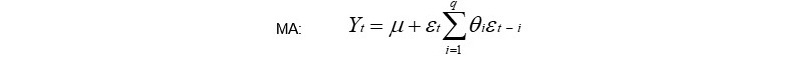
Moving averages equation.

**Figure 4:**
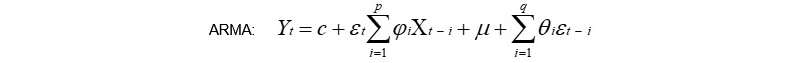
Autoregressive integrated moving average (ARIMA) equation.

As this model combines autoregression (AR) and moving averages (MA), mathematically, it can be expressed as seen in Figures 2 and 3. The combination of these models can be expressed as shown in Figure 4 where Y_t_, is the predicted value; c, is the constant; μ, is the expectation of X_t_; φ_i_.....φ_p_, and θ_i_.....θ_p_ are the parameters of each model; t, is the time; e_t_, is the white noise error terms. Generally, the easiest way to think of ARIMA models is as fine-tuned versions of random-walk and random-trend models.

#### Model Prediction for Year 2012

First, we create an estimation (base) model for the year 2011. If we assume that the parameters, the constant, and the errors of the estimate for the year 2011 are the same as for the year 2012, by downloading the values from the Google Trends, we build a model for the year 2012. This means that we tried to forecast the influenza ILI rates of 2012 having the knowledge of only the Google Trends data.

### The Case of Italy

The second method addresses the situation, when a flu score is not needed, as there is sufficient data volume of searches by Google Trends for the term *influenza*, and the correlation coefficient is above .90 (.906 for 2011 and .917 for 2012). In this case, we examined the possibility of using data from a neighboring country. We choose Italy for comparison because it is the nearest country for which we can find data from Google Trends and ECDC, and it is a close country next to Greece in the European Union.

The test case was the data for the year 2011, building a base model, as in the previous method.

The next task was to use the same parameters of the ARIMA (1, 0, 0) model (constant, φ, θ, and ε of year 2011) for the year 2012 (from the first to the last week of 2012) to develop a forecast.

As shown in the Results Section, the case of Italy is different from the Greek one because the official data from ECDC do not exist from a specific week until the end of the year 2012. Nevertheless, using the ARIMA model, we can predict the entire time series—that means the peak and the spread of influenza based on data from Google Trends.

**Figure 5:**
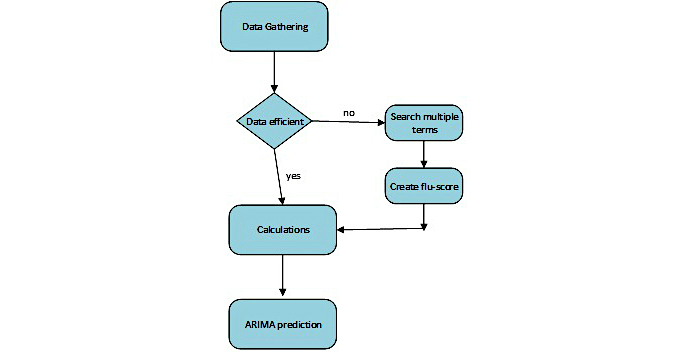
Flow diagram of methodology.

Generally, the methodology could be summarized as shown in Figure 5.

## Results

Autoregression and moving averages models may be used to correlate data and build prediction models. The methods described above were developed to address the problem of insufficient or complete missing data of Google or even the statistical correlation is below .90. This correlation is denoted by the Pearson *r* correlation coefficient.

### The Case of Greece

In the first method, combining the most relevant keywords in Greek language to the term *influenza*, we had the results for 2 years as follows:

For the year 2011, the *r* coefficient is above .90 at significance level *P*=.01 (two-tailed), and this means that the estimation is quite precise. This means that the distribution of the data gathered by Google follows the same distribution of the influenza cases notified by the public authority in Greece. Consequently, an early prediction is very accurate.

**Figure 6:**
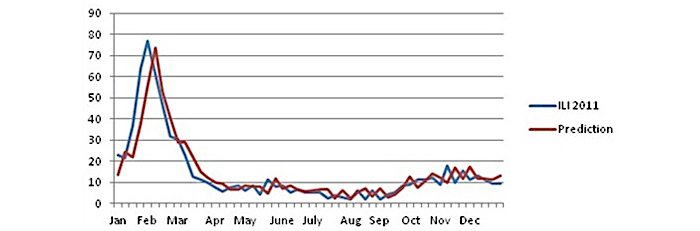
Estimation for Greece using the autoregressive integrated moving average (ARIMA) model (year 2011).

The results for the year 2011 are shown in Figure 6.

The horizontal axis in Figure 6 represents time, whereas the vertical axis represents the ILI cases of influenza. The ILI 2011 data line shows the values of the ILI data (ILI rates) from KEELPNO, which is the dependent variable. The prediction (red) line shows the estimated values calculated by the ARIMA model. As it is clear, using this model, we can obtain a very good estimation of the development of influenza in Greece for this year. The correlation (*r* coefficient) is greater than .90, and it is .909 at significance level *P*=.01.

**Figure 7:**
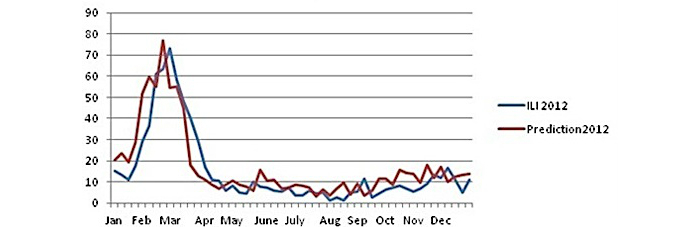
The prediction for Greece (year 2012).

Some interesting remarks can be mentioned about the above estimation:

- The predicted development of the disease is almost the same as the real one.
- The predicted peak appears with a delay of 1 week after the actual one in early February in the 6th week instead of the 5th week of the real cases.
- The predicted peak is 73.35, very close to the real peak, which is 76.98. The difference is below 5% (4.71%).
- It takes 4 weeks to reach the maximum value from the baseline, which are the 20 ILI cases (from the first week to the 5th week for the real values and from the second week to the 6th week for predicted values).
- The above estimation is very good, and we tested the same model with the same parameters to establish a prediction model for the next year.
- For the year 2012, the *r* coefficient is over .80 but below .90 at significance level *P*=.01. Nevertheless, we cannot reject the usability of the model, as we still predict the exact time of the peak of seasonal influenza (early March) and the size of the peak, as shown in Figure 7.
- The correlation coefficient (*r*) is greater than .80, and it is .831. Although it is below .90, we can still use the model.

Some interesting aspects of the forecast for the year 2012 are the following:

- The forecast of the peak of the seasonal flu is almost accurate, considering that during the year 2011 (from the first to the last week of 2011), the peak arrived very early in February (5th week), whereas in 2012 (from the first to the last week of 2012), there is a significant difference, as the peak arrived later in the 9th week.
- It takes the same time (as for the year 2011) for the peak to appear (4 weeks above the baseline of 20).
- The forecast model predicts almost accurately the year 2012. The predicted value is 76.61, and the real value is 73.26, which means that the difference is below 5% (4.58%).
- The predicted peak is shown 1 week earlier.
- The development of the curve after the peak also follows closely the observed data.
- The prediction in this case is based on that for both years the maximum activity of the disease appears exactly 4 weeks after the value comes to a point of more than 20 ILI cases, which is the baseline of influenza activity, as previously mentioned.
- The final point of the forecast will be the assumption of an early detection.

Table 2 shows the rising of influenza.

From Table 2, it is shown that the disease rises above the value of 20 in different weeks, but it takes the same time to reach the peak (9−5=4 weeks and 5−1=4 weeks). In conclusion, by using our forecast model for the year 2012, we predicted almost accurately the peak of seasonal influenza in Greece 4 weeks before it arrived, from the start of the year, based exclusively on the knowledge of the Google search queries, before the ILI rates are officially calculated by the competent authorities **.**

Finally, the comparison of real and predicted (ILI per 1000 people) cases is shown in Table 3.

### The Case of Italy

In the case of Italy, we can see that for the year 2011, the coefficient *r* is greater than .90, which means that seasonal influenza estimated value is almost the same comparing to the real value. Using the ARIMA model, the coefficient *r* remains over .90, and this indicates a statistical correlation at significance level *P*=.01 (two-tailed). That means we can use this model as a good estimation for the development of the disease.

**Figure 8:**
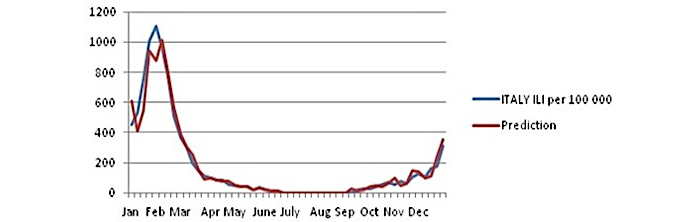
Autoregressive integrated moving average (ARIMA) model for Italy (year 2011).

The result of the ARIMA model is shown in Figure 8.

**Table 2:** Rising from 20 to maximum.

Value	Year	Week above 20	Week of the peak
>20	2012	5th	9th
>20	2011	1st	5th

**Table 3:** Prediction of the real cases for year 2012 (peak).

Predicted value	Real value	Difference	Difference (%)
76.61	73,260	3.35	4.58

The prediction of ILI rates for Italy, using the ARIMA model, is very good, and the correlation coefficient *r* is .979 at significance level *P*=.01.

The outcome of the model indicates that there is a strong and significant statistical correlation between the Google searches made by Italians for the word *influenza* and the ILI rates given by the European competent authority.

The main results of this estimation can be summarized as follows:

- The development of the disease is almost the same after the peak.
- The estimation model predicts the highest value 1 week later.
- The baseline is 500 ILI cases (per 100,000 people).
- It takes 4 weeks to reach the maximum value (1st to 5th week).
- The real maximum value is 1102.1, whereas the estimated value is 1013.05. The difference is −89.1 (−8.08%).

The prediction results for the year 2012 are as follows:

For the year 2012, the coefficient *r* is over .90 (.923), and this means that we can make a precise prediction. Using the ARIMA (1, 0, 0) model, the time of the peak of influenza is indicated in mid-February (6th week), even though with a higher value than the actual one. During the year 2011, Italian influenza peaked to the value of 1102.1. The predicted point for this year is 1037.461. The (predicted) difference is 35.361, whereas the actual difference is lower (947−1102.1=−155.1)

**Figure 9:**
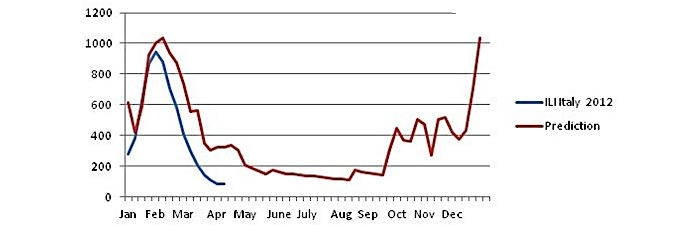
Autoregressive integrated moving average (ARIMA) model. Prediction for Italy (year 2012).

The prediction of this model for the year 2012 is shown in Figure 9.

As shown in Figure 9, the prediction for the year 2012, based on the ARIMA model parameters of the year 2011, is almost the same but with a little bit of higher values.

The statistical correlation is above .90. The correlation coefficient *r* is .923, and this means this correlation is statistically significant at a significance level of *P*=.01.

Let’s see the main results of this prediction model:

There is a lot of missing official data for ILI cases after the 16th week (mid-April).

Despite the missing values, the prediction of the peak and the size of the peak are very good. The predicted value is 1037.461, and the real peak is 947. The difference is 90.46 (9.55%). The predicted peak occurs 1 week later than the real peak (6th week instead of 5th week).

There is another peak to the end of the year, such as that of the 6th week. The value of the baseline before this peak arrives is 500 ILI cases and occurs at the end of the year starting 5 weeks earlier (46th week).

The predicted baseline is 400 ILI cases, above which it takes 4 weeks for the peak to arrive.

The summary of the results of ARIMA (1, 0, 0) model for both Greece and Italy cases is shown in Table 4.

**Table 4:** Summary of the results.

Country	Year	Predicted peak	Real peak	Difference	Difference (%)	Weeks to reach the peak
Greece	2011	73.35	76.98	−3.63	−4.71	4
Greece	2012	76.61	73.26	3.35	4.58	4
Italy	2011	1013.05	1102.1	−89.05	−8.08	4
Italy	2012	1037.461	947	90.461	9.55	4
Italy	2012	1037.461				5

## Discussion

### Prediction

Autoregression and moving averages models may be used to correlate data and build prediction models. The methods described above were developed to use the data from Google searches found in the Google Trends system with the help of ARIMA models. The first method is used when searches for the term *influenza* do not give sufficient volume data, or the correlation coefficient is below .90. In such a case, the alternative is to seek other keywords related to influenza. These refer to the main symptoms.

The early detection of a future influenza pandemic activity is a key issue for all public health authorities [31]. The rationale for direct actions is based on the prediction of a likely spread across Europe and triggering of national operational plans. Therefore, an early prediction is necessary to design and implement public health preparedness plans. In our study, we concluded that an early detection of influenza activity can be made with the help of the Internet. In the case of influenza in Greece and Italy, by setting an Internet surveillance system, we can predict the peak, the time of the peak, and the spread of influenza at least 4 weeks earlier, before influenza reaches its maximum point.

Similar researches, mentioned in the Introduction, were conducted by scientists who used Internet data to make predictions and estimations for infectious diseases. Different models were used to detect and predict the outbreak of seasonal diseases. The results of other researches were focused to various countries such as the United States, Sweden, the United Kingdom, or to the countries of Asia. Our research is the first that examines a serious infectious disease such as influenza in small countries such as Greece and Italy. We consider the ARIMA model, already used by other scientists, very effective, and we made use of it to make estimations and predictions for the spread and the peak of seasonal influenza in Greece and Italy.

### Restrictions

The main restrictions should be as follows:

- To perform analysis based on Google searches requires Google data to exist. This can be done when people can do searches on the Internet and, of course, it also requires a general extend of Internet penetration and use in the specific country. Although nowadays, Internet use has continuously risen; it is of great importance that the Internet speeds should be fast enough, and people are familiarized to the Google services.
- Another aspect is the language used. The keyword *influenza* may give enough data to perform analysis, but this can be a general rule for English-speaking countries, or even more for countries with the use of Latin language. As we mentioned in the first method, this cannot be done in countries with other languages, such as Greece. This is the main reason why we constructed a set of keywords and found their average values in the Greek language.
- The popularity and publicity regarding infectious diseases. The influenza disease can be safely used, as it is a very common disease among many countries of the world. Nevertheless, if there is a need for examination and study for another disease with less popularity, the first method will be possibly the only solution, when a researcher wishes to analyze data from Google Trends, specifically in smaller countries.
- Despite the above restrictions, it is certain, as other similar studies have shown that the Google Trends system can be safely used. In general, an Internet surveillance system can be an alternative system to the official sentinel systems for monitoring and evaluating the development of infectious diseases.
- There is a lot of discussion about the usability of Google Flu Trends, a service which was provided by Google. It has been found [32] that Google Flu Trends missed the emergence of the 2009 pandemic and overestimated the 2012 and 2013 influenza season epidemic. Google has shut down Google Flu Trends predictions, acknowledging the problem. Klembczyk et al suggests Google Flu Trends as a stand-alone surveillance system because it is most useful as an early signal system used in conjunction with other more comprehensive surveillance techniques.

### Usability to National and International Systems

The outlook of testing different systems and generally the use of Internet surveillance systems is very important. This does not mean that monitoring systems based on Internet surveillance should totally substitute the traditional systems, but they can be certainly used on a supplementary basis.

Besides the above remark, a monitoring system based on Internet data may take advantage of the same definitions, methods, and indicators created and proposed by international and national organizations. Consequently, this means that this kind of system can have a great contribution to coordinate the different national monitoring systems.

The official definitions of diseases and the proposed specific indicators are made to coordinate the national systems. In Europe, ECDC monitors the levels of influenza activity in European countries reported by EISN members during the influenza season. The levels are based on the following three assessments or indicators [33] of influenza activity: 

- An indicator of the overall intensity of influenza activity in the country
- An indicator of the geographical spread of influenza in the country
- An indicator of trend in ILI or acute respiratory infection (ARI) sentinel consultations in the country compared with the previous week

The main three indicators concern the overall intensity of influenza activity, the geographical spread of influenza, and the trend of the disease. These indicators can be described as follows:

#### Indicator of the Overall Intensity of Influenza Activity

The intensity of influenza activity is based on the overall level of clinical influenza activity in the country (or region). Each country assesses the intensity of clinical activity based on the historical data at its disposal. Some countries have historical data that date back over 30 years (eg, the United Kingdom [England] and the Netherlands), whereas others have data that date back over shorter periods of time (eg, Ireland). Some networks can establish numeric thresholds that define the different intensity levels of clinical influenza activity.

The EISN intensity definitions are denoted as low, medium, high, very high, and unknown.

The baseline influenza activity is the level that clinical influenza activity remains in throughout the summer and most of the winter. Usually, there will be a 6- to 12-week period in winter when the level of clinical influenza activity rises above the baseline threshold, but in the very occasional winter, activity never gets above the baseline level.

#### Indicator of the Geographical Spread of Influenza in the Country

Each country defines the geographical spread of influenza according to the definitions outlined below. The definitions are based on those used by the WHO global influenza surveillance system—FluNet [34].

- ILI: influenza-like illness
- ARI: acute respiratory infection
- Country: countries may be made up of one or more regions
- Region: the population under surveillance in a defined geographical subdivision of a country. A region should not (generally) have a population of less than 5 million unless the country is large with geographically distinct regions

The geographical spread is indicated through as no-activity, sporadic, local outbreak, regional activity, and widespread activity.

#### Indicator of Trend in ILI or ARI Sentinel Consultations in the Country Compared With the Previous Week

Trend is reported by the countries as increasing, stable, or decreasing. Trend is a comparison of the level of ILI or ARI sentinel consultations during 1 week with the previous week.

Outside the influenza season, when ILI and ARI rates are at baseline level, increasing or decreasing trends are not informative.

Increasing: evidence that the level of respiratory disease activity is increasing compared with the previous week.

Stable: evidence that the level of respiratory disease activity is unchanged compared with the previous week.

Decreasing: evidence that the level of respiratory disease activity is decreasing compared with the previous week.

The usability of the aforementioned definitions and indicators indicate that an Internet surveillance system may be a useful tool to manage a coordination of the different national systems that are currently used.

In terms of government spending, we mentioned in the introduction the huge costs connected to influenza through absenteeism, influenza complications, and hospital stays and deaths. We believe that early detection could provide useful means and tools for preventing purposes to reduce the overall spending but mostly to address public health issues concerning influenza tracking, monitoring, and treatment. Many studies in various universities and research centers have been conducted to indicate and propose the extensive use of the Internet to meet the requirements for a successful monitoring of epidemics and for creating an Internet surveillance system in an inexpensive way.

### Conclusions

Finally, the main conclusions of this study can be summarized as follows:

- There is a significant statistical correlation with influenza ILI rates of Greece and Italy and the searches made in Google search engine.
- We can use the ARIMA statistical model for estimations and to create prediction rules and patterns for influenza in Greece and Italy based on searches made in Google search.
- By using Google Trends, we can predict the maximum point of influenza 4 weeks before it arrives.
- Google Trends can be a useful source of data. In cases of insufficient data or with low correlation of Google searches to the real cases for a single word (influenza) for a specific location (country) and for a certain period (year), a combined flu score can be created based on Google searches made by people with keywords related to the symptoms of the disease. When sufficient and reliable data volume of a keyword exists, we can still use ARIMA models for forecasts.
- An Internet surveillance system can be an alternative, as it can operate as a supplementary system, and it can use the same official definitions and indicators of the traditional systems to help coordinating national monitoring systems across Europe.
- On the basis of Google search data, an Internet system can contribute to lowering costs by helping governments to prevent severe influenza outbreaks and manage their operational public health plans.

The term *Infodemiology* refers to information epidemiology and was first used by Gunther Eysenbach [9, 35] to propose a *new research discipline and methodology* on the study of the determinants and distribution of health information on the Internet, with the ultimate purpose to improve public health. The concept of *Infodemiology* (or *infoveillance*) is now widely used to describe the study and connection of serious disease development with the help of the Internet [21]. We believe that Google Trends could be a useful data source, which, with the help of statistics, can contribute to the abovementioned purpose by establishing an Internet surveillance system.

## Appendix

Multimedia Appendix 1 Programming codes.

## Abbreviations

AR: autoregression
ARI: acute respiratory infection
ARIMA: autoregressive integrated moving average
ARMA: autoregressive moving average
CDC: Centers for Disease Control and Prevention
ED: emergency department
EISN: European Influenza Surveillance Network
EU: European Union
ILI: influenza-like illness
KEELPNO: Hellenic Center for Disease Control and Prevention
MA: moving averages
NUTS: classification of territorial units for statistics
WHO: World Health Organization
