# Supplementary material for: Syndromic Surveillance Models Using Web Data: The Case of Influenza in Greece and Italy Using Google Trends
Source: JMIR Public Health Surveill. 2017 Nov 20;3(4):e90. doi: 10.2196/publichealth.8015 (PMC5715201; doi:10.2196/publichealth.8015)
Supplement: Multimedia Appendix 1 [file publichealth_v3i4e90_app1.pdf]

Appendix: Programming codes

1. SPSS code

```
* define a macro to do the regressions.
DEFINE !regloop(nby=!TOKENS(1)).
!DO !cnt=1 !TO !nby.
REGRESSION
   /MISSING LISTWISE
   /STATISTICS COEFF OUTS R ANOVA
   /CRITERIA=PIN(.05) POUT(.10)
   /NOORIGIN
   /DEPENDENT  Y
   /METHOD=ENTER !CONCAT('x',!cnt)
!DOEND.
!ENDDEFINE.
SET MPRINT=yes.
*Call macro.
!regloop nby=7.
```

2. Visual C# Code

```
using System;
using System.Text;
using System.Net;
using System.IO;

namespace ConsoleApplication2
{
   class Program
   {
      static void Main(string[] args)
      {
         using (var client = new WebClient())
         {
            var response =
client.DownloadString("https://www.google.com/accounts/ClientLogin?accountType=GOOGLE&Email=xxxxxxxx@gmail.com&Passwd=password&service=trendspro&source=test-test-v1");
```

```
            //var response =
client.DownloadString("https://accounts.google.com/ServiceLogin?
service=trendspro&continue=http://www.google.com/trends/&followup=http://www.go
ogle.com/trends/&Email= xxxxxxxx@gmail.com&Passwd= password &date=2012");

            // The Auth line

            var auth = response.Split('\n')[2];

            client.Headers.Add("Authorization", "GoogleLogin " + auth);

            // search terms

            var arr3 = new string[] { "γριπη", "πυρετος", "βηχας", "πονοκεφαλος",
"πονολαιμος", "φαρυγγιτιδα", "αντιβιωση" };

            var arrDownnloadData = new string[4];

            arrDownnloadData[0] = "http://www.google.com/trends/trendsReport?
hl=en-US&q=" + arr3[0] + "," + arr3[1] + "&geo=GR&content=1&export=2";

            arrDownnloadData[1] = "http://www.google.com/trends/trendsReport?
hl=en-US&q=" + arr3[2] + "," + arr3[3] + "&geo=GR&content=1&export=2";

            arrDownnloadData[2] = "http://www.google.com/trends/trendsReport?
hl=en-US&q=" + arr3[4] + "," + arr3[5] + "&geo=GR&content=1&export=2";

            arrDownnloadData[3] = "http://www.google.com/trends/trendsReport?
hl=en-US&q=" + arr3[6] + "," + "&geo=GR&content=1&export=2";

            for (int i = 0; i < 4; i++)

            {

               byte[] csv = client.DownloadData(arrDownnloadData[i]);

               Console.WriteLine(Encoding.UTF8.GetString(csv));

               //'var fileStream = File.Create("C:\report.csv");

               var fileName = "report" + i + ".csv";

               File.WriteAllBytes(fileName, csv);

            }

         }


      }

   }
```
